# Supplementary material for: Antiviral Potentialities of Chemical Characterized Essential Oils of Acacia nilotica Bark and Fruits against Hepatitis A and Herpes Simplex Viruses: In Vitro, In Silico, and Molecular Dynamics Studies
Source: Plants (Basel). 2022 Oct 28;11(21):2889. doi: 10.3390/plants11212889 (PMC9656187; doi:10.3390/plants11212889)
Supplement: Supplementary file 1 [file plants-11-02889-s001.zip › plants-1924692-supplementary.pdf]

plants

Article

Antiviral Potentialities of Chemical Characterized Essential Oils of *Acacia nilotica* Bark and Fruits against Hepatitis A and Herpes Simplex Viruses: *In-Vitro, In-Silico,* and Molecular Dynamics Studies

Supplementary Materials

Table S1: Three results of docking simulations of the major compounds identified in EOs of both bark and fruits of *Acacia nilotica*.

Compound	3C protease			TK		
Spathulenol	-5.3	-5.3	-5.1	-6.8	-6.7	-7.0
Caryophyllene oxide	-5.6	-5.5	-5.2	-6.9	-6.7	-7.3
γ-Terpinene	-4.8	-4.8	-4.8	-5.8	-5.8	-5.8
Z-Anethole	-4.7	-4.6	-4.7	-5.2	-5.2	-5.2
trans-Caryophyllene	-5.3	-5.3	-5.2	-6.58	-6.9	-6.9
